# High gain and wideband hybrid optical amplifier using bismuth-doped and neodymium-doped fibers for E-band applications

**DOI:** 10.1038/s41598-025-05507-x

**Published:** 2025-07-01

**Authors:** Aleksandr Donodin, Leily Kiani, Shabnam Noor, Wladek Forysiak

**Affiliations:** 1 Aston Institute of Photonics Technologies, Aston University, Birmingham, UK; 2 Lawrence Livermore National Laboratory, Livermore, CA 94550 USA

**Keywords:** Optics and photonics, Applied optics, Fibre optics and optical communications

## Abstract

A hybrid dual-stage bismuth-doped fiber and neodymium-doped fiber amplifier with high optical gain and extended bandwidth of operation in the E-band is demonstrated. The amplifier features a maximum gain of 43 dB, output power of 372 mW, and a minimum noise figure of 5.5 dB, and operation wavelength range of 1397–1472 nm, enabled by 153-m of bismuth-doped fiber and two 7-m lengths of neodymium-doped fiber. The demonstration shows the possibility of achieving improved gain bandwidth by combining fiber amplifiers with overlapping gain spectra in the E-band.

## Introduction

The rapid growth of network traffic due to demanding internet services such as high-definition video streaming, cloud computing and artificial intelligence, in addition to a fully occupied C-band, has led to the exploration of new approaches to increase the capacity of WDM systems and upgrade the optical network infrastructure. Methods for obtaining greater channel capacity include increasing the signal-to-noise ratio (SNR), the spectral bandwidth and the number of spatial paths. Significant gains in channel capacity can be attained by increasing the spectral bandwidth, rather than increasing the SNR, as the increase in channel capacity is linear in bandwidth, while it is logarithmic in SNR^1,2^. While capacity can also be increased by using more spatial paths, simply by deploying multiple fibers in the C-band, technologies like multi-band or ultra-wideband (UWB) transmission (via a single fiber) exhibit great potential, with UWB fulfilling many of the desired criteria in the short-to-mid-term^3,4^.

UWB transmission relies on the availability of appropriate amplification technologies and there is a need to develop new components and sub-systems in order to address new amplification bands^5,6^. The C and L bands are already well-covered by commercially available erbium doped fiber amplifiers (EDFAs), which have reached a mature stage of development and serve as a standard for gain and noise figure characteristics of other amplification technologies. Raman amplifiers^7^ and semiconductor optical amplifiers (SOAs)^4^ have been studied extensively, and in principle, can be used in any band, while other types of doped fibre amplifiers and combinations of amplifiers can serve as options for amplification bands other than C+L^4^.

Due to benefits such as availability of wide bandwidth and low propagation loss of standard single mode fiber, the S- and E-bands are a logical choice when it comes to expanding spectral coverage beyond the C+L bands. However, despite the commercial availability of S-band compatible thulium-doped fiber amplifiers (TDFAs), their use in systems has not been adopted to date, due to various issues (i.e. poor gain and NF in the shorter wavelength part of S-band) ^4^. Moreover, the performance of the C- and L-bands can be affected by the close proximity of the S-band due to inter-band stimulated Raman scattering (ISRS)^8^. On the other hand, the impact of ISRS on C- and L-bands from the E-band is lower when an appropriate guard band between them is created^9^. Moreover, the E-band has ample bandwidth (more than 3x the C-band), availability of low water peak fibers^3,10^ and a range of emerging amplification options, that include the Raman amplifiers^11^, SOAs^12^, bismuth-doped fiber amplifiers (BDFAs)^13–15^ and neodymium-doped fiber amplifiers (NDFAs)^16,17^.

Raman amplifiers are able to provide gain at any required bandwidth, subject to availability of appropriate pump wavelengths. However, the gain medium of these amplifiers has a length in the order of kilometers and there are noise sources that are unique to these amplifiers^7,11^. SOAs are also able to provide gain over broad wavelength ranges but have high noise figure and WDM signals are subject to nonlinear crosstalk^4^. BDFAs exhibit significant gain and good noise figure (NF)^13,18^ and are possibly the most attractive among all the current options in the E-band, despite their relative immaturity. Bismuth-doped fiber is similar to traditional erbium-doped fiber in terms of fiber splicing and mechanical properties. However, the gain mechanisms of bismuth ions are not yet well understood and until recently, there was no mature fabrication process^4^. Moreover, high gain per meter to realize relatively short amplifiers was not considered possible, but significant progress has been reported in recently ^18^. Neodymium doped fiber also amplifies in the E-band and provides good NF (approximately 5 dB) in designs where the challenges from competitive electronic transitions are addressed with photonic-crystal like elements to enable efficient E-band amplification^16^. These amplifiers are also unable to provide enough gain in their current stage of development but straightforward improvements in fabrication^16^ could result in improved gain performance. To date, there has been only a single demonstration of the use of NDFAs in optical coherent communications^19^.

In the recently demonstrated transmission system utilizing O-, E-, S-, C-, L-, and U-bands, amplification in the E-band was performed by BDFAs based on germanosilicate BDFA^20^. Even though, the performance of the BDFA was acceptable to support coherent transmission in the range of 1410 to 1460 nm, the fundamental limitation of germanosilicate BDFAs to target lower E-band (below 1410 nm) was highlighted. It is well known, that BDFAs based on phosphosilicate fiber can cover both O- and E-bands, however, typically require higher pump power to achieve similar output power to germanosilicate BDFAs^15,21^. Thus, here we propose an alternative approach to target amplification in lower E-band region. Germanosilicate glass based BDFAs have good performance in the long wavelength region of the E-band, while NDFAs perform best in the middle of the E-band, resulting in partially-overlapping gain spectra. Thus, we propose an alternative approach to enhance amplification in the lower E-band. Germanosilicate glass-based BDFAs perform well in the long-wavelength region of the E-band, while NDFAs provide stronger gain in the middle of the E-band, resulting in partially overlapping gain spectra. This approach shifts amplification towards lower wavelengths at the expense of S-band performance, which is typically supported by TDFAs in multi-band systems. Therefore, here we explore whether they can be combined to utilize their complementary nature in order to augment the gain bandwidth, and improve overall performance. This work explores a novel hybrid bismuth-doped fiber and neodymium doped fiber amplifier, with high optical gain and extended bandwidth of operation in the E-band. While BDFA-based hybrid doped amplifiers (E + S band hybrid bismuth/erbium) have been explored previously^22,23^, to date, there has been no demonstration of a hybrid NDFA-BDFA. The demonstrated hybrid amplifier features a maximum gain of 43 dB and a minimum noise figure of 5.5 dB, enabled by 153-m of Bi-doped fiber and 2 $\times$ 7-m lengths of Nd$^{3+}$-doped fiber.

## Experimental setup and methodology

**Fig. 1:**
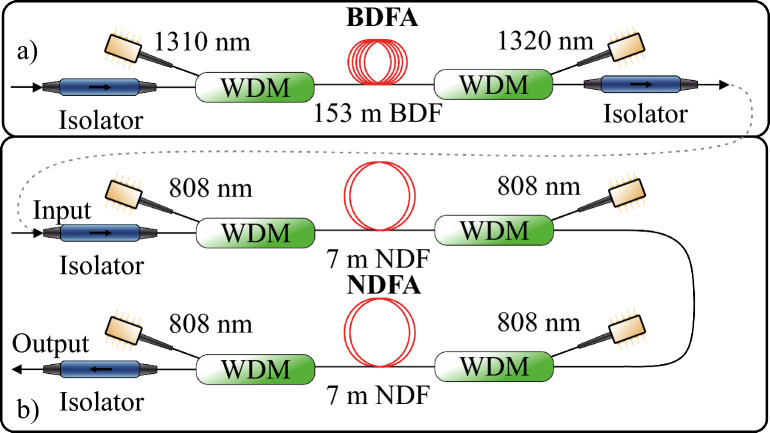
Schematics of bismuth-doped fiber amplifier (**a**) and neodymium-doped fiber amplifier (**b**). The grey dashed line represents the fiber connection when the hybrid BDFA is the pre-amplifier and the NDFA is the booster amplifier.

The schematics of the hybrid amplifier are shown in Fig. 1, with the BDFA shown in Fig. 1a and the NDFA in Fig. 1b. A set of pump diodes is used for each amplifier to allow the best performance in terms of gain and NF. The BDFA consists of an active Bi-doped fiber, two isolators for unidirectional propagation of the signal and two thin-film-filter wavelength division multiplexers (WDMs) for multiplexing signal and pump radiation. The Bi-doped germanosilicate fiber used in this work is fabricated using the conventional MCVD doping technique and has a length of 153 m. The fiber has core and cladding diameters of 6 $\upmu m$ and 125 $\upmu m$, respectively. The index difference ($\Delta n$) between the core and cladding is around 0.007. The fiber core consists of 95 mol% $SiO_2$, 5 mol% $GeO_2$ and <0.01 mol% of bismuth. The cutoff wavelength ($\lambda _c$) of the fiber is measured to be around 1100 nm.

The NDFA, shown in Fig. 1b consists of two identical amplification stages, each consisting of a 7 m-long neodymium-doped fiber, pumped bi-directionally by two 808 nm pump laser diodes. To combine signal and pump radiation, two WDMs are used in each amplification stage. Two signal isolators at the input and the output of the amplifier are used to ensure uni-directional operation. Note that an additional isolator between the stages is not required for robust operation, as it leads to increased loss between the stages, consequently decreasing the overall performance of the amplifier. Cladding pumping with high-power multimode diodes was not considered, as its interaction with the fiber filtering microstructure remains uncertain, and no effort was made to implement a second cladding during fiber fabrication. In this proof-of-concept demonstration, wavelength division multiplexers (WDMs) were chosen for their simplicity, despite their inherent losses. Future work will explore polarization beam combining as a means to improve pump absorption efficiency and reduce losses. The active neodymium-doped fiber is an all-solid micro-structure with a glass diameter of 126 $\upmu \hbox {m}$ and a micro-structure pitch of 6.6 $\upmu \hbox {m}$. The micro-structure acts as a wavelength-selective waveguide that suppresses gain between 850 and 1150 nm. The term ‘all-solid microstructure’ differentiates this fiber from conventional microstructured fibers that rely on air holes for light guidance. By using only solid glass materials to engineer its guiding properties, this fiber offers improved splicing compatibility and mechanical robustness, making it more practical for real-world applications. This is required as the E-band transition of $Nd^{3+}$ ($^4F_{3/2}$ to $^4I_{13/2}$) is significantly weaker than the transitions from $^4F_{3/2}$ to $^4I_{9/2}$ and $^4F_{3/2}$ to $^4I_{11/2}$, giving rise to unwanted lasing through amplifier spontaneous emission near 920 nm and 1100 nm, respectively^24^. The core glass is a mixture of Nd$^{3+}$-doped, fluorine-doped and pure silica components where the original Nd$^{3+}$ doped glass was co-doped with 0.75 mol $Al_2O_3$. The Nd$^{3+}$ ion concentration creates a small signal absorption of 57 dB/m at 808 nm as measured by Optacore SA from a slice of the glass rod. This amplifier design has been described previously^16^, but here, we use a higher total pump power of 1.4 W from four diodes to achieve higher net gain.

**Fig. 2:**
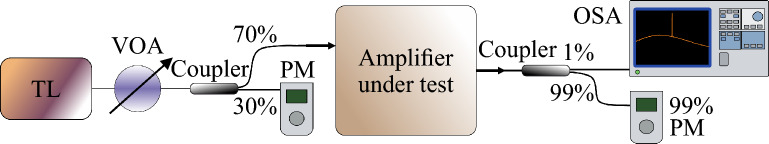
Schematic of gain and NF measurement. *TL* tuneable laser, *PM* power meter, *OSA* optical spectrum analyzer.

A schematic of the experimental setup used for gain and noise figure (NF) measurements is presented in Fig. 2. The tuneable laser (TL) is used to produce a signal with high SNR (>70 dB) in the spectral range from 1355 to 1485 nm. The input signal power $P^{in}_{PM}$ is monitored via a 30% coupler output using a power meter (PM), and the variable optical attenuator (VOA) is controlled to set the required input signal power to the amplifier. After the signal is amplified it is split with a coupler, where 99% goes to another PM to record output power $P^{out}_{PM}$, and 1% goes to the optical spectrum analyzer (OSA).

To determine the optical gain G, the amplified signal power $P^{out}_{s}$ is calculated from the total output power $P^{out}_{PM}$, taking into account that it makes up a fraction *f* of the total output power spectrum measured at the PM, which also includes unabsorbed pump radiation and amplified spontaneous emission noise. Moreover, the contribution of the output coupler includes its coupling ratio $R_{out}$. The wavelength dependent gain $G_{lin}(\lambda )$ is obtained as the ratio of the signal output power $P^{out }_{s}$ to its input power, $P^{in}_{s}$,

1 $$\begin{aligned} G(\lambda ) = 10\cdot log_{10}\left( \frac{P^{out}_{PM}(\lambda )\cdot f(\lambda )\cdot R_{out}(\lambda )}{P^{in}_{PM}(\lambda )\cdot R_{in}(\lambda )}\right) = 10\cdot log_{10}\left( G_{lin}(\lambda )\right) , \end{aligned}$$

where $P^{in}_{s}$ is determined with readings of the corresponding PM ($P^{in}_{PM}$), taking into account the coupling ratio of the input coupler, $R_{in}$.

The noise figure (NF) is determined using the noise subtraction technique^25^:

2 $$\begin{aligned} NF(\lambda ) = 10\text{ log}_{10}\left( \frac{\rho _{noise}(\lambda ) }{G_{lin}(\lambda )h\nu }+\frac{1}{G_{lin}(\lambda )}\right) , \end{aligned}$$

where $\rho _{noise}$ is the noise spectral density at the output of the amplifier, *h* is Planck’s constant, and $\nu$ is the frequency of the signal radiation. The input noise is negligible as compared to other sources of noise due to a high signal-to-noise ratio at the input ($>70$ dB). The spectral noise level at the signal wavelength is approximated from the optical spectrum, and the noise spectral density is calculated taking into account the total output power $P^{out }_{PM}$ measured by PM. The proposed characterization method is estimated to have gain and NF accuracy of 0.2 dB of two standard deviations. Finally, the power conversion efficiency (PCE) is determined using the following formula:

3 $$\begin{aligned} PCE(\lambda ) = \frac{P^{out}_{PM}(\lambda )\cdot f(\lambda )\cdot R_{out}(\lambda )-P^{in}_{PM}(\lambda )\cdot R_{in}(\lambda )}{P^{pump}}, \end{aligned}$$

where $P^{pump}$ is the total pump power of the amplifier.

**Fig. 3:**
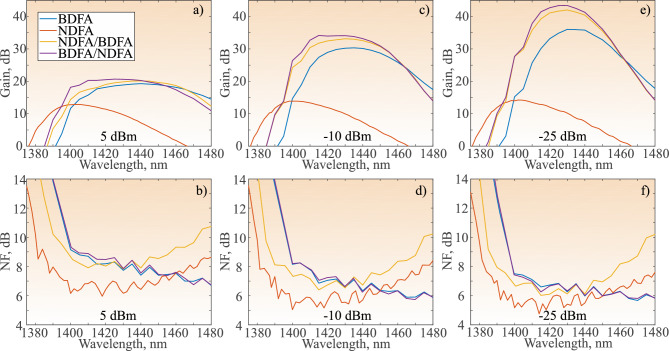
Spectra of the recorded gain for the four amplifiers with input signal powers of 5 dBm (**a**), − 10 dBm (**c**), − 25 dBm (**e**); and spectra of recorded NF for the four amplifiers with input signal powers of 5 dBm (**b**), − 10 dBm (**d**), − 25 dBm (**f**).

## Amplifier performance

All the amplifiers are characterized in terms of gain, output power, and NF at three levels of input signal power: 5 dBm, − 10 dBm, and − 25 dBm. The graphical comparison of gain and NF spectra for all signal powers and amplifiers is presented in Fig. 3.

First, the BDFA performance is analyzed with the total bi-directional pump power of 1 W and presented in Fig. 3 in blue. The gain peak of the BDFA is around 1430 nm and it reaches 19.3 dB, 30.3 dB, and 36 dB at 5 dBm, − 10 dBm, and − 25 dBm input signal power, respectively. It can be seen that the gain decreases significantly from lower (Fig. 3e) to higher input power (Fig. 3a). Along with the gain saturation, the gain flatness decreases significantly. The range of operation, as well as the main amplifier parameters and results obtained are presented in Table 1. The NF of the BDFA decreases gradually with increase in wavelength, leading to the minimum NF in the S-band. The lowest NF in the E-band of 6 dB is achieved at 1450 nm. The NF increases significantly with increase in input signal power, reaching 6.2 dB and 7 dB at − 10 dBm and 5 dBm. Maximum output power of 269 mW is achieved with 5 dBm input signal power.

**Table 1:** Comparison of the main amplifier characteristics of the different amplifiers. Range of operation is determined as follows: 3-dB bandwidth for a 5 dBm input signal power , and a 20 dB gain for − 10 dBm and − 25 dBm input signal power. The NDFA range of operation is always determined by 3 dB bandwidth.

$P_{in}$, dBm	Amplifier	Gain, dB	NF, dB	Operation range, nm	$P_{out}$, mW	PCE, %
	BDFA	36	5.5	1406–1475	12.6	1.3
− 25	NDFA	14.2	4.8	1389–1424	0.08	0.006
	NDFA/BDFA	42	6	1397–1471	50	2
	BDFA/NDFA	43.4	5.5	1397–1472	69.2	2.9
	BDFA	30.3	5.8	1406–1474	107	11
− 10	NDFA	13.9	5.1	1389–1426	2.4	0.17
	NDFA/BDFA	33.1	6.3	1398–1471	204	8.5
	BDFA/NDFA	34.2	5.7	1397–1471	263	10.9
	BDFA	19.3	7	1411–1471	269	27
5	NDFA	12.9	6	1388–1429	62	4
	NDFA/BDFA	20.1	7.8	1407–1467	324	13
	BDFA/NDFA	20.7	7	1399–1458	371	15.4

Next, the NDFA is characterized with 1.4 W total pump power, with the power uniformly distributed among the four pump laser diodes in both stages, at 0.35 W each. The recorded gain and NF for this case are presented in red in Fig. 3. The gain of the NDFA decreases slightly with increase in input signal power (Fig. 3a,c,e). The NDFA exhibits constant gain flatness, leading to an almost constant 3-dB range of operation (refer to Table 1) However, this effect leads to a much smaller maximum gain achieved by the NDFA: 12.9 dB, 13.9 dB, and 14.2 dB for 5 dBm, − 10 dBm, and − 25 dBm input signal power, respectively. This effect of slow gain saturation shows a great potential for high power applications. Compared to the BDFA, the bandwidth of operation of the NDFA is shifted towards the shorter E-band. The NDFA shows significantly lower NF in the whole E-band compared to BDFA, but rises quickly in the S-band. The minimum achieved NF is presented in Table 1. A minimum value of 4.8 dB is again achieved at − 25 dBm input signal power at 1414 nm. The complementary nature of the NDFA and BDFA spectra suggests a wider amplification bandwidth compared to the individual amplifiers could be achieved with an amplifier hybrid^22^.

**Fig. 4:**
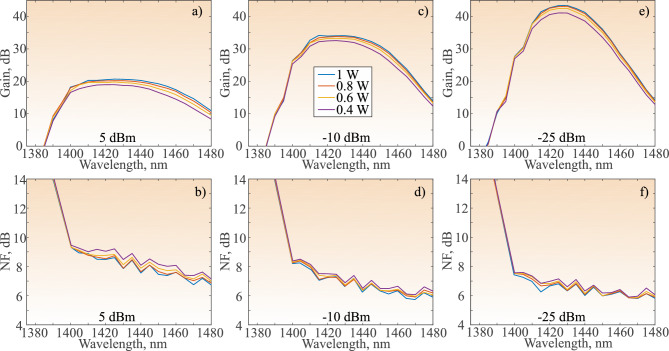
Spectra of the recorded gain for four pump powers of BDFA/NDFA amplifier with 5 dBm (**a**), − 10 dBm (**c**), − 25 dBm (**e**) input signal power; and spectra of recorded NF for four pump powers of BDFA/NDFA amplifier with 5 dBm (**b**), − 10 dBm (**d**), − 25 dBm (**f**) input signal power.

Following the performance characterization of the individual amplifiers, the two hybrid amplifier designs are characterized. The first hybrid configuration is the NDFA/BDFA, in which the NDFA functions as a pre-amplifier and is followed by the BDFA, which functions as a booster amplifier. The gain and NF spectra for different input signal powers for the NDFA/BDFA configuration are presented in Figure 3 in yellow. The gain spectra for all three input signal powers shift towards the shorter wavelength range in the E-band. The achieved maximum gain is higher compared to both BDFA and NDFA at all wavelengths and is summarized in Table 1. On the other hand, the gain in the S-band (1465 nm) is lower. This spectral shift can be explained by the shape of the NDFA gain spectra and its loss above 1465 nm. The range of operation of this amplifier lies wholly within the E-band, compared to the BDFA, where the longer wavelength part of gain is pronounced in the S-band. The achieved NF (Figures 3,b,d,f) is better below 1420 nm, compared to the BDFA for all wavelengths. However, the overall NF performance is worse in the whole spectral range, compared to the NDFA. Moreover, the NF at wavelengths longer than 1430 nm rises quickly, due to the higher NF of the pre-amplifier in this range (NDFA).

Finally, the hybrid BDFA/NDFA amplifier configuration is characterized, in which the BDFA acts as the pre-amplifier while the NDFA acts as the booster amplifier. The results of gain and NF measurements are presented in purple in Fig. 3. The maximum gain of 20.7 dB, 34.2 dB, and 43.4 dB is achieved with 5 dBm, $-10$ dBm, and $-25$ dBm input signal power, respectively. As above, the gain peak is shifted towards 1420 nm, similar to the NDFA/BDFA case. The operation range for this amplifier lies more in the E-band, compared to BDFA and NDFA/BDFA cases, where the longer wavelength gain is pronounced in the S-band. By sacrificing the gain in the S-band, a significant increase in the short wavelength side of the E-band is achieved. For example, for $-25$ dBm input signal power the operation bandwidth is 1397–1472 nm, for $-10$ dBm it is 1397–1471 nm, and for 5 dBm it is 1399–1458. Overall, the operation bandwidth improvement in the E-band ranges from 9 to 12 nm, when compared to the BDFA. The NF of the BDFA/NDFA is almost the same as the NF of the BDFA, due to the fact that BDFA mainly determines the overall NF of the BDFA/NDFA hybrid amplifier, as it is used as a pre-amplifier. The achieved NF values along with other parameters are outlined in Table 1.

Output power of all amplifiers is also compared for three different input signal powers. An obvious conclusion that can be made, is that NDFA has a very subtle change in gain with input signal power, leading, to abrupt increase of the output power from 0.08 mW to 62 mW for $-25$ dBm and 5 dBm input signal powers, respectively. This behavior demonstrates its viability as a power amplifier. In contrast, output power of BDFA shows a subtle growth with input power. Even though, both hybrid amplifiers show good output power (higher than 300 mW for 5 dBm input signal power), the BDFA/NDFA outperforms the other amplifiers and achieves highest output power for all input signal powers with maximum of 371 mW for 5 dBm input signal power.

As the final amplifier performance metric, the PCE is compared. For 5 dBm input signal power, the BDFA/NDFA PCE of 15.4% outperforms all other amplifiers except the BDFA. At $-10$ dBm input signal power, the PCE of the BDFA/NDFA reaches almost the same level as the BDFA with 11%. At a small signal power of $-25$ dBm, the BDFA/NDFA PCE outperforms all amplifiers with 2.9% PCE. This effect can be explained by the interplay between the BDFA’s ability to provide very high gain at small signal powers, and the NDFA’s ability to provide low gain saturation at high input signal powers.

**Table 2:** Comparison of the main amplifier characteristics for different pump powers of the pre-amplifier in the BDFA/NDFA amplifier. Operation range is determined as follows: 3-dB bandwidth for 5 dBm input signal power, and 20 dB gain for $-10$ dBm and $-25$ dBm input signal power.

$P_{in}$, dBm	Amplifier	Gain, dB	NF, dB	Operation range, nm	$P_{out}$, mW	PCE, %
	1 W	43.4	5.5	1397–1472	69.2	2.9
-25	0.8 W	43.2	5.7	1397–1471	66.1	3
	0.6 W	42.5	5.9	1397–1470	56.2	2.8
	0.4 W	41.1	6	1397–1469	40	2.2
	1 W	34.2	5.7	1397–1471	263	10.9
-10	0.8 W	33.8	5.9	1397–1470	239.9	11
	0.6 W	33.3	6	1397–1469	213.8	10.6
	0.4 W	32.6	6.1	1398–1467	182	10
	1 W	20.7	7	1399–1458	372	15.4
5	0.8 W	20.2	7.1	1399–1458	331	15
	0.6 W	19.8	7.2	1400–1456	302	15
	0.4 W	19	7.4	1399–1453	251.2	13.9

As the BDFA/NDFA design exhibits the most favorable overall performance and good potential for expanding the bandwidth towards the lower E-band, the performance of this design is characterized with different pump powers in the pre-amplifier stage. Decreasing the pump power in the booster stage results in a decline in performance and thus, the results are not presented in this work. The pump power could not be increased due to the limitations of the pump lasers. The compared pump powers are 0.4 W, 0.6 W, 0.8 W, and 1 W of the BDFA (pre-amplifier) stage. The graphical comparison of the measured spectra of the gain and NF at input signal powers of 5 dBm, $-10$ dBm, and $- 25$ dBm are presented in Fig. 4. Decreasing the pump power predominantly impacts the peak and longer wavelength part of the gain spectra due to the BDFA operating in that spectral range. The outline of the main parameters for the different pump powers is presented in Table 2. At 5 dBm input signal power, the maximum gain gradually decreases from 20.7 dB at 1 W pump power to 19 dB at 0.4 W pump power. On the other hand, the NF of the amplifier increases from 7 dB to 7.4 dB at 1W and 0.4 W pump powers, respectively. The operation bandwidth does not decrease dramatically until 0.4 W, where it decreases to 1399–1453 nm from 1399–1458 nm at 0.8 W and 1 W. The PCE gradually decreases with the decrease of pump power from 15.4% for 1W to 15% for 0.8 W and 0.6 W, before sharply falling further to 13.9%. At $-10$ dBm and $-25$ dBm input signal power gain, NF, anc operation bandwidth behave in the similar manner with pump power change as for 5 dBm input signal power. However, unlike the 5 dBm case, a decrease of pump power from 1 W to 0.8 W leads to the PCE rise to 11% from 10.9% and to 3% from 2.9% for the $-10$ dBm and $-25$ dBm input signal power, respectively. A further decrease of the pump power, decreases the PCE.

## Discussion

It should be noted, that the individual amplifiers were not designed specifically to work in a hybrid design. The BDFA and NDFA were initially designed as independent amplifiers and later combined into a hybrid system. As a result, two isolators, with a loss of only 0.3 dB each, were retained between the stages. Moreover, the following combinations and optimized performance may differ if the BDFA and NDFA stages are specifically designed to work together. However, the main findings above serve as a first step and are indicative towards the optimal design. The proposed use of two amplifiers stages with different spectral characteristics offers certain tunability due to the fact that optical gain spectra are overlapping. Thus, the resultant gain spectra somewhat differs from the position of each amplifier. However, because the designed NDFA works best with higher input-signal power by providing low gain saturation, it is better suited to be the booster amplifier. On the other hand, the BDFA provides the best gain for low input signal powers, and functions best as the pre-amplifier in this particular case. This resembles the classical dual-stage design of erbium-doped fiber amplifiers, where the pre-amplifier provides high gain, and the booster provides high output power. We would like to emphasize that both amplifiers were not specifically designed to perform in a dual-stage process, and that, for instance, the NF of the BDFA can be significantly improved as shown in^21^. With correct optimization and management, further improvements in gain and NF operation can be achieved, especially if the S-band performance can be sacrificed.

This work presents a BDFA/NDFA design that shifts the gain spectrum by 12 nm into the E-band compared to a standalone BDFA (from 1411-1471 nm to 1399-1458 nm). While this extension may appear limited, in a DWDM system, it provides an additional 1.8 THz of usable bandwidth, supporting 28 extra 64 GBaud channels and increasing fiber capacity by approximately 14 Tbit/s with DP-16-QAM modulation. Although further optimization of the NDFA could enhance short-wavelength gain, this is fundamentally restricted by excited-state absorption (ESA), which is dependent on the glass host material. Exploring alternative host compositions could mitigate ESA but was beyond the scope of this study.

Directly comparing PCE and operational bandwidth across different studies is challenging, as these metrics are often reported under varying signal power conditions, which can obscure a direct one-to-one comparison. However, based on available data we note that reference^15^reports a PCE of 3.5% for an input signal of -10 dBm in the E-band, whereas our hybrid BDFA/NDFA achieves a significantly higher PCE of 11% under similar conditions. While the amplifier in reference^15^ shows a broad 116 nm gain bandwidth for small-signal amplification, its gain drops below 20 dB between 1375-1390 nm due to residual OH absorption, potentially limiting its applicability. Reference^14^ demonstrates a high PCE of 22% at 38 mW input power, decreasing to  15% at 3 mW (5 dBm), which is comparable to our proposed amplifier. However, the gain profile in the reference^14^ is highly dependent on both signal and pump powers, making its performance less predictable. Reference^21^ reports a PCE of 38% for a pure germanosilicate fiber-based BDFA, but its gain rapidly degrades at wavelengths below 1410 nm, while also providing strong amplification in the S-band. This limits its effectiveness for targeted lower E-band amplification. Moreover, our dual-stage BDFA/NDFA design provides higher overall gain, improving signal amplification beyond levels reported in previous works, while also ensuring a more favorable gain profile for amplification in the E-band.

## Conclusion

This work demonstrates the development and characterization of a hybrid bismuth-doped fiber and neodymium-doped fiber dual-stage amplifier. Two different design scenarios of the amplifier were studied: with the BDFA as a pre-amplifier and the NDFA as a booster (BDFA/NDFA), and vice versa (NDFA/BDFA). Better performance in terms of range of operation and gain was achieved by the BDFA/NDFA design, compared to the individual amplifiers and to the NDFA/BDFA design. The developed amplifier reaches a maximum gain of 43.4 dB, maximum output power of 69.2 mW, a minimum NF of 5.5 dB, and operation bandwidth between 1397-1472 nm for small signal amplification, and a maximum PCE of 15.4% and output power of 372 mW for 5 dBm input signal power.

## Data Availability

Data underlying the results presented in this paper are not publicly available at this time but may be obtained from A.D. (a.donodin@aston.ac.uk) upon reasonable request.
